# Productive performance of Mexican Creole chickens from hatching to 12 weeks of age fed diets with different concentrations of metabolizable energy and crude protein

**DOI:** 10.5713/ab.20.0682

**Published:** 2020-12-11

**Authors:** Miguel Ángel Matus-Aragón, Fernando González-Cerón, Josafhat Salinas-Ruiz, Eliseo Sosa-Montes, Arturo Pro-Martínez, Omar Hernández-Mendo, Juan Manuel Cuca-García, David Jesús Chan-Díaz

**Affiliations:** 1 Program of Animal Science, College of Postgraduates, Campus Montecillo, Texcoco, State of Mexico 56230, Mexico; 2 Department of Animal Science, Chapingo Autonomous University, Chapingo, State of Mexico 56230, Mexico; 3 College of Postgraduates Campus Cordoba, Cordoba, Veracruz 94946, Mexico; 4 Trouw Nutrition Mexico, S.A. de C.V., Zapopan, Jalisco 45150, Mexico

**Keywords:** Apparent Metabolizable Energy, Carcass Yield, Crude Protein, Mexican Creole Chickens, Nutrient Utilization, Productive Performance

## Abstract

**Objective:**

The study aimed to evaluate the productive performance, carcass yield, size of digestive organs and nutrient utilization in Mexican Creole chickens, using four diets with different concentrations of metabolizable energy (ME, kcal/kg) and crude protein (CP, %).

**Methods:**

Two hundred thirty-six chickens, coming from eight incubation batches, were randomly distributed to four experimental diets with the following ME/CP ratios: 3,000/20, 2,850/19, 2,700/18 and 2,550/17. Each diet was evaluated with 59 birds from hatching to 12 weeks of age. The variables feed intake (FI), body weight gain (BWG), feed conversion (FC), mortality, carcass yield, size of digestive organs, retention of nutrients, retention efficiency of gross energy (GE) and CP, and excretion of N were recorded. Data were analyzed as a randomized block design with repeated measures using the GLIMMIX procedure of SAS, with covariance AR (1) and adjustment of degrees of freedom (Kendward-Roger), the adjusted means were compared with the least significant difference method at a significance level of 5%.

**Results:**

The productive performance variables BWG, mortality, carcass yield, fat and GE retention and excretion of N were not different (p>0.05) due to the diet effect. In the 3,000/20 diet, the chickens had lower values of FI, FC, crop weight, gizzard weight, retention, and retention efficiency of CP (p<0.05) than the chickens of the 2,550/17 diet.

**Conclusion:**

The Mexican Creole chickens from hatching to 12 weeks of age can be feed with a diet with 2,550 kcal ME and 17% CP, without compromising productive parameters (BWG, mortality, carcass yield) but improving retention and retention efficiency of CP.

## INTRODUCTION

The production of Mexican Creole chickens provides animal protein and income to families in rural communities [1]. Birds of this genotype can survive and produce meat and eggs even with inadequate nutrition and unsanitary conditions [2]. Although Mexican Creole birds are an important genetic resource, they have been little studied and their nutritional requirements such as metabolizable energy (ME) and crude protein (CP) are unknown. Studying the retention efficiency of ME and CP is key in animal nutrition given that those nutrients represent approximately 90% of the total cost of the diet in domestic chickens [3]. Additionally, an imbalance in these components in the diet can retard growth [4] and reduce economic returns.

Feed intake (FI) in chickens is influenced by the concentra tion of ME in the diet. When diets are low in ME, FI increases. But if the feed is bulky and exceeds the storage capacity of the digestive system, the chickens may not consume adequate amounts of nutrients [4]. On the contrary, if the concentration of ME is high in relation to CP and amino acids, fat deposition can increase [5,6] and body weight gain (BWG) can be compromised [4]. Excess CP in diet can results in excess of N excretion in litter house causing skin dermatitis, footpad lesions and hock burns and ammonia emission [7].

Although there are few studies on the feeding of the Mexican Creole chickens using diets with different concentrations of ME and CP, the specific nutritional needs of this genotype of birds are unknown. Some examples of these studies are: Segura-Correa et al [8], used a diet with 3,098 kcal ME/kg and 21% CP from 0 to 21 days of age and a diet with 2,998 kcal ME/kg and 19% CP, from 22 to 49 days of age. Mata-Estrada et al [1], used diets with 3,000 kcal ME/kg and 19% CP from 0 to 18 days and a diet with 2,800 kcal ME/kg and 18% CP from 19 to 177 days of age. Therefore, the aim of this study was to evaluate the productive performance, carcass yield, size of digestive organs and nutrient utilization in Mexican Creole chickens, using four diets with different concentrations of ME and CP, in order to have an estimation of the requirements of ME and CP in this genotype of birds.

## MATERIALS AND METHODS

The experiment was carried out from January to July 2019, in the poultry facilities of the Colegio de Postgraduados, Campus Montecillo, in Texcoco, State of Mexico, Mexico, at coordinates 19° 29′ N, 98° 54′ W, and an altitude of 2,247 m.

### Chickens and management

Two hundred thirty-six straight-run chicks, coming from eight incubation batches, were randomly distributed to four experimental diets. Each diet was evaluated with 59 birds (30 males and 29 females) from hatching to 12 weeks of age. At hatching, the chickens were individually identified with marks on the interdigital membranes, according to what was established by Storey [9]. From hatching to eight weeks of age, the birds were housed in electric brooders (0.40×1.10×0.40 m) with an initial temperature of 32°C, which was gradually reduced to 28°C. Later and until 12 weeks of age, the birds were housed in pens of 1.0×1.5×1.0 m, with a bed of wood shavings and an average room temperature of 24°C. Water and feed were provided *ad libitum* throughout the experimental period. The chickens were cared according to the guidelines established by the Animal Welfare Committee of the Colegio de Postgraduados, Campus Montecillo, State of Mexico, Mexico.

### Experimental diets

Four diets were formulated with different concentrations of ME and CP (Table 1), maintaining constant ratios of 150 ME (kcal/kg) and CP (%): 3,000/20; 2,850/19; 2,700/18; and 2,550/17. The ingredients used in the formulation of the diets were analyzed with the NIRS foss model DS2500 equipment (Hilleroed, Denmark). Requirements of the essential amino acid, calcium and phosphorus were met according to the NRC [10] for broilers.

### Productive performance

The productive performance variables: FI (g/chicken), BWG (g/chicken), feed conversion (FC; g/g) were recorded weekly, and mortality daily.

### Carcass yield

At 12 weeks of age, the weight of the carcass and its parts were determined, as well as the corresponding yields, according to Van Harn et al [7]. In each of the eight blocks (incubation batches), two birds were randomly selected per experimental diet, in total 16 birds per diet were obtained (eight males and eight females). Chickens were slaughtered according to the Official Mexican Standard NOM-033-SAG/ZOO-2014 [11], using a stunner electric knife followed by slaughtering and bleeding.

### Digestive organs and abdominal fat

In addition to carcass yield variables data were also collected on relative empty weight of the crop, proventriculus, gizzard, small intestine and caeca, relative weight of liver, pancreas and abdominal fat, and relative length of the small intestine and caeca, according to Mera-Zúñiga et al [12]. These variables were expressed in relation to body weight.

### Nutrient utilization

At 12 weeks of age, the chemical composition (moisture, dry matter, CP, fat, ash and gross energy [GE]), nutrient retention (CP, fat, and GE), GE and CP retention efficiency were determined for the whole-body of chickens, as well as excretion of N, according to the methodology described by Aletor et al [5]. Two birds were randomly selected per experimental diet in each block, in total 16 birds per diet (eight males and eight females).

The birds were subjected to a 12-hour of fast before they were slaughtered. The slaughter of the birds was performed using a stunner electric knife and cervical dislocation, while avoiding loss of blood. The whole-body of each chicken was frozen at −20°C. Subsequently, these were thawed and placed in an autoclave for 5 hours at 110°C and a pressure of 1 atm. Finally, each chicken body was individually placed in an industrial blender for 10 minutes and a sample of 300 g of ground meat was lyophilized and analyzed for chemical composition in order to estimate the values of nutrient retention, and retention efficiency of GE and CP. Analyzes of the chemical composition of the body were performed in triplicate, according to the AOAC [13]. The GE or heat of combustion was determined using an isoperibolic calorimeter (No. 1266, Parr instruments, Moline, IL, USA). The procedures described above to determine the chemical composition of the whole-body of the birds were also performed for a sample of 16 chickens at hatching.

Nutrient retention was estimated according to the follow ing expression:

CP retained (g)=CP in the whole body of the chicken at 12 weeks of age-average CP in the whole body of chicks at hatching

Likewise, fat and GE retained were calculated, substitut ing in the previous expression, the CP value for the fat (g) or GE (kcal) value, respectively.

The retention efficiency of GE and CP were calculated as follows:

$$\begin{array}{l} \text{GE retention efficiency }(\%)=\frac{\text{kcal GE retained}}{\text{kcal GE consumed}}\times 100\\ \text{CP retention efficiency }(\%)=\frac{\text{g CP retained}}{\text{g CP consumed}}\times 100 \end{array}$$

Nitrogen excretion was calculated as follows:

N excretion (g)=N consumed-N retained in the whole body of the chicken at 12 weeks of age

### Statistical analysis

Data were analyzed according to a randomized block design with repeated measures using the GLIMMIX procedure of SAS version 9.4 (SAS Institute, 2013) [14], AR (1) covariance structure and Kenward-Roger degrees of freedom adjustment [15]. Statistical differences were established at p<0.05 and adjusted means were compared with the least significant difference method. For the variables of productive performance and nutrient retention efficiency, the effect of diets was studied; while for the variables of carcass yield, size of digestive organs, chemical composition of the whole-body and nutrient retention, the effects of diet and sex of the chickens, as well as their interaction, were studied.

## RESULTS

### Productive performance

In the period from hatching to 12 weeks of age, differences (p<0.05) were detected among diets for the FI and FC variables. In contrast, BWG and mortality were not different (p> 0.05) among the diets (Table 2). The FI was highest (p<0.05) in the 2,550/17 and 2,700/18 diets, followed by the 2,850/19 and 3,000/20 treatments. Feed conversion was lower (p<0.05) in the 3,000/20 diet compared to the 2,700/18 and 2,550/17 diets.

### Carcass yield

The diet had a significant effect (p<0.05) only on the weight of the wings (Table 3). The sex of the birds also affected (p< 0.05) the variables studied, except for legs and thigh yield. However, there was no diet×sex interaction (p>0.05) for any of the variables evaluated. The weight of the wings was significantly higher (p<0.05) in chickens fed the diet 2,550/17 compared to the diet 3,000/20. Male chickens had higher (p<0.05) weight and performance in most of the variables studied, except in breast and wing yields, which were higher (p<0.05) in females.

### Digestive organs and abdominal fat

Diets affected (p<0.05) the relative weight of the crop, gizzard and pancreas (Table 4). Sex had an effect (p<0.05) on the relative empty weight of the proventriculus, gizzard and caeca, relative weight of liver and pancreas, and relative length of the small intestine and caeca. The relative weight of the crop was higher (p<0.05) in chickens fed diets 2,850/19 and 2,550/17 compared to those fed diet 3,000/20. The relative weight of the gizzard was higher (p<0.05) in the chickens fed diet 2,550/17 compared to the other diets. The relative weight of the pancreas was higher (p<0.05) in chickens fed diet 2,550/17 compared to chickens fed diet 2,850/19. Female chickens had a greater (p<0.05) weight of the proventriculus, gizzard, caeca, liver and pancreas, as well as a greater length of the small intestine and caeca, compared to male chickens.

### Nutrient utilization

Chemical composition of the whole-body of the chickens was not affected (p>0.05) by diet, sex or the interaction (Table 5). Diet had a significant effect (p<0.05) on CP retention, and sex affected (p<0.05) CP and fat retention (Table 6). Chickens fed 3,000/20 diet had lower (p<0.05) CP retention compared to the other three diets, and male chickens had higher (p< 0.05) CP and fat retention than female chickens.

A lower (p <0.05) CP retention efficiency was observed in chickens fed diet 3,000/20 compared to the other diets (Table 7). In contrast, the GE retention efficiency was lower (p<0.05) in the chickens fed the two diets with lower levels of ME and CP (2,700/18 and 2,550/17). Nitrogen excretion tended to be lower (p<0.0961) in chickens fed diet 2,550/17 compared to those on the diet 3,000/20, but differences were not statistically significant.

## DISCUSSION

The Mexican Creole chickens are an unexplored genetic resource, so most of the variables evaluated in this study were compared with the results of investigations carried out in Creole and commercial chickens.

The present investigation showed that BWG of Mexican Creole chickens from hatching to 12 weeks of age was not affected by decreasing of ME and CP in the diet from 3,000 kcal ME/kg and 20% CP to 2,550 kcal ME/kg and 17% CP, keeping the levels of essential amino acids constant. However, FI and feed conversion increased. These results are consistent with other investigations [5,16], where it was found that broilers fed with diets low in ME and CP can maintain BWG because FI increased. Likewise, Leeson and Summers [4] reported that by reducing ME in commercial poultry diets, FI increased to meet the chicken energy needs, which would explain the differences in feed conversion observed in this work.

Carcass yield was not affected by the experimental diets. In general, males showed higher weights and carcass yields than females. Probably these results are due to the constant levels of essential amino acids in the diets, particularly lysine and methionine, because these two amino acids are mainly involved in the formation of muscle tissue [17,18].

The diets with a lower concentration of ME and CP allowed include more fibrous ingredients. There are some investigations that showed that moderate fiber inclusion in diets improves the development and functions of the gizzard [19, 20], increases the secretion of HCl, bile acids and enzymatic secretions from the pancreas [21]. This in turn improves the gastroduodenal reflux that facilitates the contact between nutrients and digestive enzymatic secretions. The improved contact increases the relative weight of crop, proventriculus and gizzard [22]. All the above mentioned may explain why chickens, fed with the diet lower in ME and CP, showed greater relative weight of the crop, gizzard and pancreas. Mabelebele et al [23] reported that digestive organs in male chickens are heavier and longer than in females, which can result in greater production of digestive enzymes and a greater contact surface for the absorption of nutrients, and result in a higher rate of growth. In the present experiment, the females had greater relative empty weight of the proventriculus, gizzard and caeca, greater relative weight of the liver and pancreas as well as greater relative length of the small intestine and caeca. This could be due to a lower value of the denominator when the relative weight was estimated [12,24].

The chemical composition of the whole-body of chickens did not change by effect of the diet. This was probably due to the fact that a ratio of 150 (kcal ME kg/% CP) was kept constant in the experimental diets. It has been observed that increasing this ratio (>172) induces a higher rate of lipogenesis, which changes the chemical composition of the chicken body [5]. With the diet 3000/20, less CP retention was observed. This is consistent with Belloir et al [25], who reported lower retention of N in the body of male Ross PM3 chickens, when they were fed with diets with high CP content. In the present study, probably the energy of the diet was higher than that required by the birds, which resulted in a lower FI. In agreement with Leeson and Summers [4], birds fed the 3,000/20 diet consumed fewer grams of CP compared to the others.

Females had lower retention of CP and fat, due to the fact that they had a lower live weight compared to males. That is, the content of CP and body fat varied due to the differences in body weight that result from the sexual dimorphism of chickens [26]. Some research reported that females had lower rates of CP deposition and higher fat deposition [27].

Chickens fed diets 2,700/18 and 2,550/17 had lower GE retention efficiency, which could be interpreted as a better balance of ME, CP, and amino acids, that decreased the availability of nutrients for storage via the catabolism of amino acids to form glycogen [25]. The CP retention efficiency was lower in chickens fed diet 3,000/20, which agrees with Belloir et al [25], who observed lower N retention with diets high in CP due to deamination of amino acids, lower CP retention efficiency, and increasing N excretion [4].

In conclusion, it is possible to feed the Mexican Creole chickens from hatching to 12 weeks of age with a diet of 2,550 kcal ME/kg and 17% CP, without affecting BWG, and carcass yield, but improving the retention and retention efficiency of CP. These ME and CP values can be used as a reference point for the design of diets for this genotype of birds.

## Figures and Tables

**Table 1:** Composition (%) and calculated analysis of the experimental diets used

Items	ME/CP concentrations of diets			

	3,000/20	2,850/19	2,700/18	2,550/17
Ingredients (%)				
Maize	55.682	56.743	52.119	49.513
Soybean meal	24.037	19.827	14.899	14.154
Yellow corn DDGS	6.000	6.000	6.000	5.701
Canola meal	6.000	6.000	6.000	5.701
Soybean oil	2.944	1.001	0.502	0.476
Wheat bran	2.000	6.894	16.736	15.898
Calcium carbonate	1.317	1.328	1.332	1.264
Dicalcium phosphate	0.910	0.913	0.907	0.861
Mineral-vitamin premix^1)^	0.502	0.502	0.502	0.476
Sodium chloride	0.307	0.263	0.211	0.200
DL-Methionine	0.117	0.139	0.165	0.157
L-Lysine	0.069	0.173	0.288	0.274
Sodium bicarbonate	0.063	0.122	0.192	0.184
L-Threonine	0.052	0.095	0.147	0.141
Oat straw^2)^	0.000	0.000	0.000	5.000
Calculated analysis (%)				
Metabolizable energy (kcal/kg)	3,000.00	2,850.00	2,700.00	2,550.00
Crude protein	20.00	19.00	18.00	17.00
Energy:protein ratio	150.00	150.00	150.00	150.00
Dry matter	88.80	88.60	88.60	88.80
Crude fiber	3.20	3.70	4.50	6.20
Calcium	1.00	1.00	1.00	1.10
Available phosphorus	0.45	0.45	0.45	0.44
Lysine	1.08	1.05	1.05	1.05
Methionine	0.47	0.40	0.40	0.41
Methionine+cystine	0.80	0.82	0.80	0.80
Threonine	0.75	0.78	0.75	0.75
Tryptophan	0.28	0.28	0.28	0.19

ME, metabolizable energy; CP, crude protein; DDGS, dried distillery grains with solubles.

1) Provided the following per kilogram of diet: vitamin A, 12,000 IU; vitamin D_3_, 1,000 IU; vitamin E, 60 IU; vitamin K, 5.0 mg; vitamin B_2_, 8.0 mg; vitamin B_12_, 0.030 mg; pantothenic acid, 15 mg; niacin, 50 mg; folic acid, 1.5 mg; choline, 300 mg; biotin, 0.150 mg; thiamine, 3.0 mg. Fe, 50.0 mg; Zn, 110 mg; Mn, 100 mg; Cu, 12.0 mg; Se, 0.3 mg; I, 1.0 mg.

2) Oat straw was used as an inert filler in diet.

**Table 2:** Cumulative productive performance of Mexican Creole chickens from hatching to 12 weeks of age fed diets with different concentrations of ME and CP

Variable	ME/CP concentrations of diets				SEM	p-value

	3,000/20	2,850/19	2,700/18	2,550/17		
Feed intake (g/chick)	3,761.43^c^	4,114.92^b^	4,359.07^a^	4,450.11^a^	71.14	<0.0001
Body weight gain (g/chick)	1,096.54	1,148.97	1,133.09	1,095.15	34.28	0.6006
Feed conversion (g/g)	3.50^c^	3.72^bc^	3.95^ab^	4.14^a^	0.10	0.0006
Mortality (%)	8.13	2.58	10.00	3.33	7.51	0.1524

ME, metabolizable energy; CP, crude protein; SEM, standard error of the means.

a–c Means with different superscripts within each row indicate differences (p<0.05).

**Table 3:** Carcass yield of 12-week-old of Mexican Creole chickens fed diets with different concentrations of ME and CP

Variable	ME/CP concentrations of diets				SEM	Sex		SEM	p-value		
		
	3,000/20	2,850/19	2,700/18	2,550/17		Male	Female		Diet	Sex	Diet×sex
Body weight (g)	1,061.39	1,151.83	1,116.80	1,158.46	38.19	1,250.31^a^	993.93^b^	27.12	0.0710	<0.0001	0.5700
Carcass weight (g)	693.33	754.09	725.94	767.62	27.97	839.02^a^	631.47^b^	19.78	0.1370	<0.0001	0.7940
Carcass yield (%)	65.32	65.47	65.00	66.26	1.24	67.10^a^	63.53^b^	0.89	0.9250	0.0026	0.5000
Breast weight (g)	167.56	173.95	174.44	186.86	8.38	191.66^a^	159.74^b^	5.22	0.1240	0.0002	0.6830
Breast yield (%)	24.17	23.07	24.03	24.34	0.67	22.84^b^	25.30^a^	0.47	0.1010	0.0012	0.4570
Leg weight (g)	106.03	119.24	109.32	121.41	4.45	131.34^a^	96.66^b^	3.18	0.5710	<0.0001	0.6970
Leg yield (%)	15.29	15.81	15.06	15.82	0.29	15.65	15.31	0.20	0.3300	0.3180	0.9260
Thigh weight (g)	107.36	119.65	114.18	121.15	6.72	131.52^a^	99.66^b^	4.80	0.2040	0.0002	0.7370
Thigh yield (%)	15.48	15.87	15.73	15.78	1.04	15.68	15.78	0.73	0.9170	0.5140	0.8140
Wings weight (g)	87.14^b^	97.03^ab^	91.00^ab^	98.02^a^	2.95	104.05^a^	82.55^b^	2.07	0.0380	<0.0001	0.8470
Wings yield (%)	12.57	12.87	12.54	12.77	0.24	12.40^b^	13.07^a^	0.17	0.9180	0.0260	0.5540

ME, metabolizable energy; CP, crude protein; SEM, standard error of the means.

a,b Means with different superscripts within each row indicate differences (p<0.05).

**Table 4:** Organs size of 12-week-old of Mexican Creole chickens fed diets with different concentrations of ME and CP

Variable	ME/CP concentrations of diets				SEM	Sex		SEM	p-value		
		
	3,000/20	2,850/19	2,700/18	2,550/17		Male	Female		Diet	Sex	Diet×sex
Relative empty weight (g/kg body weight)											
Crop	4.53^c^	6.03^a^	4.87^bc^	5.47^ab^	0.41	5.22	5.24	0.28	0.0480	0.6390	0.3020
Proventriculus	4.67	4.81	4.50	4.69	0.25	4.39^b^	4.93^a^	0.18	0.3540	0.0090	0.2750
Gizzard	22.32^b^	21.94^b^	22.71^b^	26.13^a^	1.01	20.74^b^	25.72^a^	0.71	0.0002	<0.0001	0.8310
Small intestine	18.03	18.94	18.60	18.70	1.34	17.59	19.50	0.93	0.6500	0.7400	0.5120
Caeca	4.28	4.00	4.07	4.09	0.24	3.87^b^	4.34^a^	0.17	0.7520	0.0410	0.5570
Relative weight (g/kg body weight)											
Liver	23.49	24.04	22.82	23.12	1.10	22.46^b^	24.25^a^	0.77	0.8950	0.0100	0.3420
Pancreas	2.53^ab^	2.24^b^	2.42^ab^	2.94^a^	0.21	2.35^b^	2.72^a^	0.15	0.0100	0.0150	0.9520
Abdominal fat	1.96	1.47	1.80	1.33	0.70	1.74	1.54	0.49	0.9120	0.8120	0.9500
Relative length (cm/kg body weight)											
Small intestine	102.43	104.02	105.19	100.70	5.31	93.76^b^	111.89^a^	3.75	0.6320	<0.0001	0.5500
Caeca	11.49	11.92	11.75	11.93	0.53	10.59^b^	12.91^a^	0.37	0.2970	<0.0001	0.2360

ME, metabolizable energy; CP, crude protein; SEM, standard error of the means.

a–c Means with different superscripts within each row indicate differences (p<0.05).

**Table 5:** Whole-body chemical composition of 12-week-old of Mexican Creole chickens fed diets with different concentrations of ME and CP

Variable	ME/CP concentrations of diets				SEM	Sex		SEM	p-value		
		
	3,000/20	2,850/19	2,700/18	2,550/17		Male	Female		Diet	Sex	Diet×sex
Moisture (g/kg)	658.90	647.37	630.17	639.37	20.71	633.15	654.75	16.36	0.7087	0.2433	0.8303
DM (g/kg)	341.10	352.63	369.83	360.63	20.71	366.85	345.25	16.36	0.7087	0.2433	0.8303
CP (g/kg)	220.78	232.56	249.95	246.69	12.75	243.88	231.12	9.97	0.2514	0.2700	0.4473
Fat (g/kg)	64.94	59.27	60.69	52.51	8.40	63.76	54.94	6.30	0.7084	0.2753	0.3627
Ash (g/kg)	29.73	28.20	31.06	31.00	3.86	31.84	28.15	2.98	0.9326	0.3000	0.2722
GE (kcal/kg of DM)	4,786.01	4,972.10	4,735.51	4,564.85	117.75	4,757.56	4,771.67	82.38	0.151	0.907	0.3216

ME, metabolizable energy; CP, crude protein; SEM, standard error of the means; DM, dry matter; GE, gross energy.

**Table 6:** Nutrient retention in the whole body of 12-week old of Mexican Creole chickens fed diets with different concentrations of ME and CP

Variable	ME/CP concentrations of diets				SEM	Sex		SEM	p-value		
		
	3,000/20	2,850/19	2,700/18	2,550/17		Male	Female		Diet	Sex	Diet×sex
CP retention (g/chick)	191.57^b^	267.23^a^	288.37^a^	256.97^a^	14.83	289.76^a^	212.32^b^	9.94	0.0018	<0.0001	0.3220
Fat retention (g/chick)	56.98	68.45	66.33	53.51	8.31	73.66^a^	48.97^b^	5.69	0.5597	0.0096	0.2056
GE retention (kcal/chick)	4,716.73	4,903.51	4,666.11	4,494.65	118.23	4,688.88	4,701.62	82.96	0.1493	0.9160	0.3013

ME, metabolizable energy; CP, crude protein; SEM, standard error of the means; GE, gross energy.

a,b Means with different superscripts within each row indicate differences (p<0.05).

**Table 7:** Retention efficiency of CP and GE in the whole-body of Mexican Creole chickens of 12 weeks of age fed diets with different concentrations of ME and CP

Variable	ME/CP concentrations of diets				SEM	p-value

	3,000/20	2,850/19	2,700/18	2,550/17		
CP retention efficiency (%)	25.46^b^	34.18^a^	36.75^a^	33.97^a^	2.48	0.0032
GE retention efficiency (%)	29.77^a^	29.75^a^	26.58^b^	25.22^b^	0.74	0.0005
Nitrogen excretion (g/chick)	88.42	85.50	86.89	81.06	1.94	0.0961

ME, metabolizable energy; CP, crude protein; SEM, standard error of the means; GE, gross energy.

a,b Means with different superscripts within each row indicate differences (p<0.05).
